# Transient effects of multi‐infusion ketamine augmentation on treatment‐resistant depressive symptoms in patients with treatment‐resistant bipolar depression – An open‐label three‐week pilot study

**DOI:** 10.1002/brb3.1674

**Published:** 2020-07-03

**Authors:** Chuanjun Zhuo, Feng Ji, Hongjun Tian, Lina Wang, Feng Jia, Deguo Jiang, Ce Chen, Chunhua Zhou, Xiaodong Lin, Jingjing Zhu

**Affiliations:** ^1^ Department of Psychiatry School of Mental Health Jining Medical University Jining China; ^2^ Department of Psychiatry and Imaging‐Genetics and Co‐morbidity (PNGC‐Lab) Tianjin Anding Hospital Tianjin Mental Health Center Mental Health Teaching Hospital Tianjin Medical University Tianjin China; ^3^ Department of Psychiatry Wenzhou Seventh People's Hospital Wenzhou China; ^4^ Department of Pharmacy First Hospital of Hebei Medical University Shijiazhuang China

**Keywords:** bipolar disorder, global functional connectivity density, ketamine, transient effect, treatment‐resistant depression

## Abstract

**Introduction:**

While the psychiatric benefits of ketamine have been verified through clinical trials, there is limited information about ketamine augmentation in patients with treatment‐resistant bipolar depression (TRBPD). Hence, in the present study, we investigate the therapeutic efficacy and functional brain alterations associated with multi‐infusion ketamine augmentation in patients with TRBPD.

**Methods:**

The present three‐week study included 38 patients with TRBPD, all of whom received a series of nine ketamine injections over the study period. The Hamilton Depression Rating Scale (HAMD) was used to assess the effects of multi‐infusion ketamine combined with mood stabilizers. Brain function was evaluated by global functional connectivity density (gFCD).

**Results:**

Adjunctive treatment with multiple infusions of ketamine, when combined with a mood stabilizer, could effectively alleviate depressive symptoms for one week, yet the symptoms began to relapse during the second week. Functional brain alterations were detected via gFCD. Specifically, gFCD reductions were mainly found in the bilateral insula, right caudate nucleus, and bilateral inferior frontal gyrus, while increased gFCD was mainly located in the bilateral postcentral gyrus, subgenual anterior cingulate cortex, bilateral thalamus, and cerebellum. Although gFCD alterations were sustained for up to three weeks after the first ketamine infusion, the antidepressant effects of ketamine augmentation sharply declined from the end of the second week of treatment.

**Conclusions:**

Multi‐infusion ketamine augmentation can rapidly alleviate depressive symptoms in patients with TRBPD. The clinical effects were primarily visible in the first week after treatment and partially sustained for two weeks; however, the therapeutic effects and related functional brain alterations sharply decreased from the end of the second week. Based on these findings, we demonstrated that the clinical efficacy and functional brain alterations induced by ketamine augmentation are transient.

## 1 INTRODUCTION

Treatment‐resistant depressive symptoms (TRDS), which are commonly found among patients with chronic bipolar disorder (BPD), have been associated with increased morbidity and disability among such patients (Baldessarini, Salvatore, et al., 2010; Baldessarini, Vieta, Calabrese, Tohen, & Bowden, 2010; Goodwin & Jamison, 2007; Tondo & Baldessarini, 2014; Tondo, Vázquez, & Baldessarini, 2014). The patients with bipolar disease who present with treatment‐resistant depressive symptoms are diagnosed with treatment‐resistant bipolar depression (TRBPD; Halaris, Cantos, Johnson, Hakimi, & Sinacore, 2020). Although consensus guidelines and experts have advocated for the use of monotherapy among patients with BPD, adjunctive therapy is considered for patients who relapse during maintenance treatment (Baldessarini, 2013; Baldessarini, Pérez, Salvatore, Trede, & Maggini, 2014; Tondo et al., 2014). In clinical practice, poor treatment responses are prevalent in patients with BPD, especially for bipolar depression, and providers frequently rely upon a variety of untested treatment combinations (Baldessarini, Henk, Sklar, Chang, & Leahy, 2008; Centorrino, Ventriglio, Vincenti, Talamo, & Baldessarini, 2010). These patients are diagnosed with TRBPD (Dell'Osso et al., 2009; Erfurth, Michael, Stadtand, & Arolt, 2002; González‐Isasi et al., 2010; Pacchiarotti et al., 2009; Parikh, LeBlanc, & Ovanessian, 2010).

Several studies have investigated treatment strategies for TRBPD, such as adjunctive antipsychotics. For example, McIntyre et al. reported that cariprazine is highly effective in treating depressive symptoms in patients with BPD (Earley et al., 2019; Zhuo, Tian, et al., 2019), while other studies have indicated that clozapine can improve bipolar depression and reduce the risk of suicide among these patients (Zhornitsky et al., 2010). Aripiprazole and quetiapine augmentation can also improve TRBPD (Ahn et al., 2011; Kemp et al., 2007; Ketter, Wang, Chandler, Culver, & Alarcon, 2006). However, antipsychotics are associated with several severe adverse effects, such as prolongation of the QT interval, dyslipidemia, and seizures. Additionally, only 30%–50% of patients with TRBPD experience significant improvements in depressive symptoms from antipsychotic therapies. Hence, there is an urgent need for novel pharmacological strategies in the treatment of TRBPD (Hui Poon, Sim, & Baldessarini, 2015; Li, Tang, Wang, & de Leon, 2015; Tondo et al., 2014; Undurraga et al., 2012).

In the last decade, researchers have demonstrated that ketamine can partially alleviate depressive symptoms in patients with treatment‐resistant depression (TRD; Andrade, 2017). Both structural and functional brain alterations have been observed following ketamine treatment in some patients with TRD (Abdallah, Salas, et al., 2015; Bergfeld et al., 2018; Bobo et al., 2016; Vasavada et al., 2016). More notably, several recent studies have shown that ketamine augmentation can be used to treat depressive symptoms in patients with BPD (Dodd, Fernandes, & Dean, 2015; Grunebaum et al., 2017; Lee, Della Selva, Liu, & Himelhoch, 2015; López‐Díaz, Fernández‐González, Luján‐Jiménez, Galiano‐Rus, & Gutiérrez‐Rojas, 2017; McCloud et al., 2015; Rybakowski, Permoda‐Osip, & Bartkowska‐Sniatkowska, 2017; van Wissen et al., 2017). In addition, several studies have described the strong antidepressant effects associated with ketamine augmentation, which may be related to ketamine‐induced structural or functional brain alterations (Zheng et al., 2018). However, most of the studies have focused on the immediate antidepressant effects of ketamine augmentation, while very few studies have assessed the longer‐term antidepressant effects over 3–4 weeks (Gałuszko‐Węgielnik et al., 2019; Grady, Marsh, Tenhouse, & Klein, 2018; Grunebaum et al., 2017; López‐Díaz et al., 2017; Zheng et al., 2018).

Compared to the substantial number of studies focused on ketamine‐induced brain alterations in patients with unipolar depression (Chen et al., 2019; McMillan et al., 2019; Morris et al., 2020; Nugent et al., 2019; Zacharias et al., 2020), only a few studies have investigated the functional brain alterations associated with ketamine augmentation in patients with TRBPD. Recently, Diazgranados et al. conducted a 2‐week study to investigate the antidepressant effects of ketamine infusions in patients with TRBPD and found that 71% of patients with TRBPD responded to ketamine augmentation (Diazgranados et al., 2010). The authors found that ketamine produced the highest effect on the second day, yet the antidepressant effect of a single ketamine infusion could last for up to three days. After replicating the study mentioned above, Zarate et al. also showed that a single ketamine infusion could produce therapeutic effects for up to 3 days (Zarate et al., 2012). More notably, Phillips and colleagues conducted a randomized controlled trial to investigate the use of single, repeated, and maintenance ketamine infusions for treatment‐resistant depression (Phillips et al., 2019). In the two‐week study, only 59% of participants treated with multiple injections of ketamine responded to the treatment. An effective response was defined as having a ≥50% reduction in Montgomery‐Åsberg Depression Rating Scale (MADRS) scores. The authors found that patients with treatment‐resistant depression required a median of three infusions before achieving an effective response.

To improve the treatment strategies for patients with TRBPD, more studies are needed to determine the relative long‐term clinical effects and the corresponding disease‐specific components of global functional magnetic resonance imaging (fMRI) signal and connectivity (Zheng et al., 2018). In return, this will aid in the advent of novel biomarkers to improve the diagnostics and treatment of TRBPD (Kraus et al., 2020). Previous studies have adopted fMRI to assess the functional brain alterations associated with the use of ketamine (Bryant et al., 2019; Gass et al., 2019; Maltbie, Kaundinya, & Howell, 2017; Reed et al., 2019; Sterpenich et al., 2019). Simultaneously, several studies have shown that global functional connectivity density (gFCD), which is a pivotal index to assess brain function (Reed et al., 2019), is commonly altered in patients with unipolar depression and bipolar disorder (Bryant et al., 2019; Li et al., 2019; Liu et al., 2018; Shi, Tong, Zhang, & Gao, 2019; Tomasi et al., 2010; Zhuo, Zhou, et al., 2019). FCD mapping is a voxel‐wise, data‐driven method for testing the density distribution of whole‐brain resting‐state functional connectivity, especially gFCD. The gFCD can reflect the ability of the brain to communicate information (Zuo et al., 2012). According to Thompson et al., the gFCD may represent a potential biomarker of quantitative state changes in glucose metabolism (Thompson et al., 2016).

Therefore, in the present pilot, open‐label, multi‐infusion ketamine infusion study, we aimed to investigate the therapeutic effects and associated brain alterations associated with multi‐infusion ketamine augmentation in patients with TRBPD. Hence, we adopted gFCD to examine changes in brain function after ketamine plus antidepressant treatment in patients with TRBPD. We hypothesize that: (a) Multiple infusions of ketamine may alleviate the depressive symptoms associated with TRBPD, (b) ketamine augmentation may induce functional brain alterations in patients with TRBPD, and (c) the clinical effects of ketamine augmentation may be related to the functional brain alterations.

## 2 METHODS

### 2.1 Patients

The present study was approved by the Ethics Committee of Tianjin Anding Hospital and Wenzhou Seventh People's Hospital (ZS2017011 and 15JCYBJC50800). Each participant was informed regarding the purpose and details of the experiment. Written informed consent was obtained from each patient. We recruited 60 right‐handed patients, consisting of 38 men and 22 women, who were admitted to Tianjin Anding Hospital from December 2017 to December 2019. The patients ranged from 25 to 55 years of age, were diagnosed with BPD, based on the DSM‐IV classification, and met the criteria for TRBPD. TRBPD is defined as having failed attempts to achieve remission after 8 weeks each of two or more separate monotherapies or one monotherapy with one combination treatment (Halaris et al., 2020).

The patients were mentally capable of providing informed consent. Informed consent was obtained only after a team of physicians explained the procedures for the study. In this pilot study, the patients were required to have received failed two trials of antidepressant treatment with a mood stabilizer or antipsychotic agent, without alleviation of their depression symptoms. In addition, a score of ≥24 on the 17‐item Hamilton Depression Scale (HAMD‐17) and Young Mania Rating Scale (YMRS) score of ≤5 were required for study admission. Patients had to be clinically stable on either a mood stabilizer and/or antipsychotic medication before entering the study (Hidalgo‐Mazzei et al., 2019; Lv et al., 2019). The exclusion criteria eliminated the following candidates: (a) individuals with severe physical illnesses, such as atherosclerosis, diabetes, hypertension, infection, or epilepsy; (b) women who were pregnant or planning to become pregnant during the study period; (c) individuals with any other major axis I disorders; (dd) individuals with a history of electroconvulsive therapy (ECT) in the past 6 months; (e) contraindications for MRI examination; (f) history of unconsciousness for more than 5 minutes due to any cause; (g) current suicidal ideation; and (h) IQ < 80.

### 2.2 Intravenous injections of ketamine

Ketamine dosages were calculated based on the methods of previous studies (Bonnet, 2015; Furuya et al., 1999; Papolos et al., 2018), considering the potential influence of fluctuations in norepinephrine (NE) levels. A single dose of 0.5 mg/kg ketamine was intravenously injected within 40–50 minutes of 10:00 p.m. on the second day after entering the study. Subsequently, ketamine was administered at the same time on days 4, 6, 8, 10, 12, 14, 16, 18, and 20. During and after each intravenous injection, electrocardiography (ECG) was performed, and blood pressure and oxygen levels were monitored for 1 hr. HAMD scores were recorded each week after ketamine augmentation.

### 2.3 Mood stabilizer and antidepressant therapy

In the present study, patients could continue their current treatment with mood stabilizers, such as sodium valproate, atypical antipsychotics, and lithium. In addition, all patients were receiving antidepressant therapy with agents such as lamotrigine (an agent commonly used for bipolar disorder maintenance therapy or augmentation therapy in depression), sertraline, citalopram, bupropion, and others. Moreover, due to the patients having treatment‐resistant disease, almost all of them were administered antipsychotic agents and benzodiazepines, such as quetiapine or clonazepam, as a synergist to alleviate the symptoms of the disease, although they did not acquire the optimal treatment effect. Each patient had received treatment with two or more antidepressants at adequate dosages for at least 12 weeks. All patients received prior treatment with benzodiazepines, such as clonazepam and diazepam. Currently, there is limited information about calculating the dose equivalents for antidepressants in patients with depression (Hayasaka et al., 2015). Due to the lack of an appropriate conversion formula, it was not possible to determine the uniform equivalent doses of antidepressants and diazepam. However, to account for this limitation, we fixed all therapeutic agents for the duration of the ketamine infusions.

### 2.4 Acquisition of MRI data

The trajectory of ketamine augmentation was characterized following the methods described in the literature (Li et al., 2019). Briefly, patients underwent five MRI scans, the first of which was performed prior to beginning treatment with ketamine. Additional scans were performed on the 2nd, 7th, 14th, and 21st days after initiating ketamine treatment. MR images were acquired using a 3T GE Discovery MR750 scanner (General Electric) equipped with an eight‐channel phased‐array head coil. During the scanning sessions, the participants were required to remain still while in the supine position, without falling asleep. A gradient‐echo echo‐planar imaging sequence with the following parameters was used to acquire whole‐brain resting‐state fMRI (rs‐fMRI) data depicting the blood‐oxygen‐level‐dependent (BOLD) signal: repetition time (TR) = 2,000 ms; echo time (TE) = 45 ms; slices = 32; slice thickness = 4 mm; gap = 0.5 mm; field of view (FOV) = 220 × 220; matrix size = 64 × 64; and flip angle (FA) = 90°. Parallel imaging using the sensitivity encoding (SENSE) technique was used to acquire all images (SENSE factor: 2). A high‐resolution 3D Turbo‐Fast Echo T1WI sequence was used to obtain structural images, with the following parameters: 188 slices, TR/TE = 8.2/3.2, slice thickness = 1 mm, no gap, FA = 12°, matrix size = 256 × 256, and FOV = 256 × 256 (Bryant et al., 2019; Gass et al., 2019; Li et al., 2019; Liu et al., 2018; Reed et al., 2019; Shi et al., 2019; Sterpenich et al., 2019; Tomasi et al., 2010; Zhuo et al., 2014; Zhuo, Zhou, et al., 2019).

### 2.5 Data preprocessing

SPM8 was used to process the resting‐state fMRI data (http://www.fil.ion.ucl.ac.uk/spm). To allow for imaging unit stabilization and patient familiarization, the first ten volumes of scans were discarded. The remaining volumes were corrected for slice timing and motion artifacts. Translational and rotational head movements were less than 2 mm and 2° for all participants, respectively. Covariates, including head motion, white matter signal, and cerebrospinal fluid signal, were regressed out from the time series of every voxel. Here, the Friston 24‐parameter model was used to regress out the effects of head motion. Next, the frame‐wise displacement (FD) was calculated, and the data were regressed out when the FD of a specific volume was >0.5. The datasets were filtered with band‐pass frequencies ranging from 0.01 to 0.08 Hz. Individual structural images were coregistered to the mean functional image, and the transformed structural images were coregistered to the Montreal Neurological Institute (MNI) space via linear registration. The motion‐corrected functional volumes were spatially normalized to the MNI space using parameters estimated during the linear coregistration. Finally, the functional images were resampled into 3‐mm cubic voxels for further analysis (Bryant et al., 2019; Gass et al., 2019; Li et al., 2019; Liu et al., 2018; Shi et al., 2019; Sterpenich et al., 2019; Reed et al., 2019; Tomasi et al., 2010; Zhuo et al., 2014).

### 2.6 Calculation of gFCD

The gFCD of each voxel was calculated using an in‐house Linux script, as previously described (Zhuo et al., 2014). Functional connectivity between the voxels was evaluated using Pearson's linear correlation with a correlation coefficient threshold of *R* > .6 (Liu et al., 2018; Zhuo et al., 2014). The gFCD calculations were limited to those voxels within the cerebral gray matter mask, and the gFCD at any given voxel (x0) was calculated as the total number of functional connections [k(x0)] between x0 and all other voxels using a growth algorithm, which was repeated for all x0 voxels. Next, gFCD was divided by the mean value of the qualified voxels in the brain to increase the normality of the distribution. The gFCD maps were spatially smoothed using a 6 × 6 × 6 mm^3^ Gaussian kernel to minimize differences in the functional anatomy of the brain between patients.

### 2.7 Statistical analysis

Changes in gFCD were corrected using family‐wise error (FWE). Changes in HAMD scores and gFCD were compared using paired *t* tests. Spearman's correlation coefficients were used to examine the association between changes in HAMD scores and gFCD. The level of statistical significance was set at *p* < .05.

## 3 RESULTS

**FIGURE 1:**
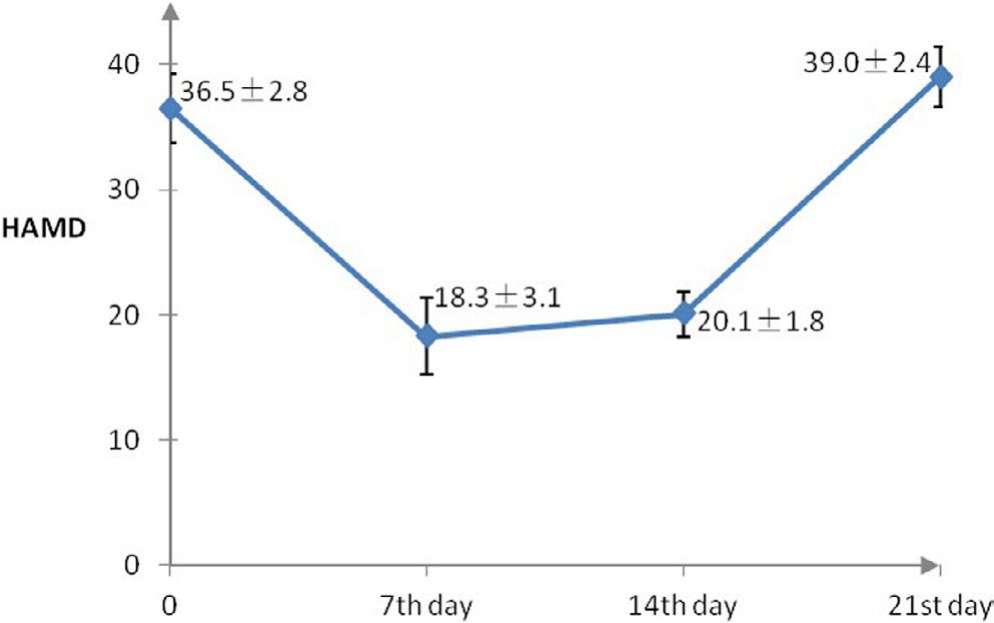
The trajectory of the antidepressant effect of treatment with multi‐infusion ketamine augmentation over three weeks

We excluded data for nine patients due to poor image quality (*n* = 5) and the lack of follow‐up MRI data (*n* = 4). In addition, 13 patients who stopped ketamine treatment due to side effects were also excluded from this study. Hence, we analyzed the MRI data of 38 patients, including 22 men and 16 women. Significant differences in HAMD scores were observed after one week of ketamine plus antidepressant treatment, with an average reduction of 49.8% (baseline score: 36.5 ± 2.8 vs. day 7 score: 18.3 ± 3.1; *p* < .05; Table 1, Figure 1). However, patients began to experience relapses of depressive symptoms during the second week. Surprisingly, by 21 days after starting treatment, the patients reported depressive symptoms that were more severe than before starting ketamine treatment (baseline HAMD score: 36.5 ± 2.8 vs. day 21 score: 39.0 ± 2.4; Figure 1).

**TABLE 1:** The characteristics and clinical data of patients included in this study (*n* = 38)

	Sample	*t*	*p‐*Value
Gender (*n*)	Male (22)	1.428	.011
	Female (16)		
Age (years)	43.10 ± 5.3		
Education (years)	15.8 ± 5.9		
Duration of illness (months)	100.5 ± 23.2		
HAMD score before ketamine plus antidepressant treatment	36.5 ± 2.8	22.175	.000
HAMD score on day 7 after initiating treatment	18.3 ± 3.1		
HAMD scores after 3 weeks of treatment	39.0 ± 2.4		
YMRS scores before ketamine plus antidepressant treatment	0		
YMRS scores after ketamine plus antidepressant treatment	0		

**FIGURE 2:**
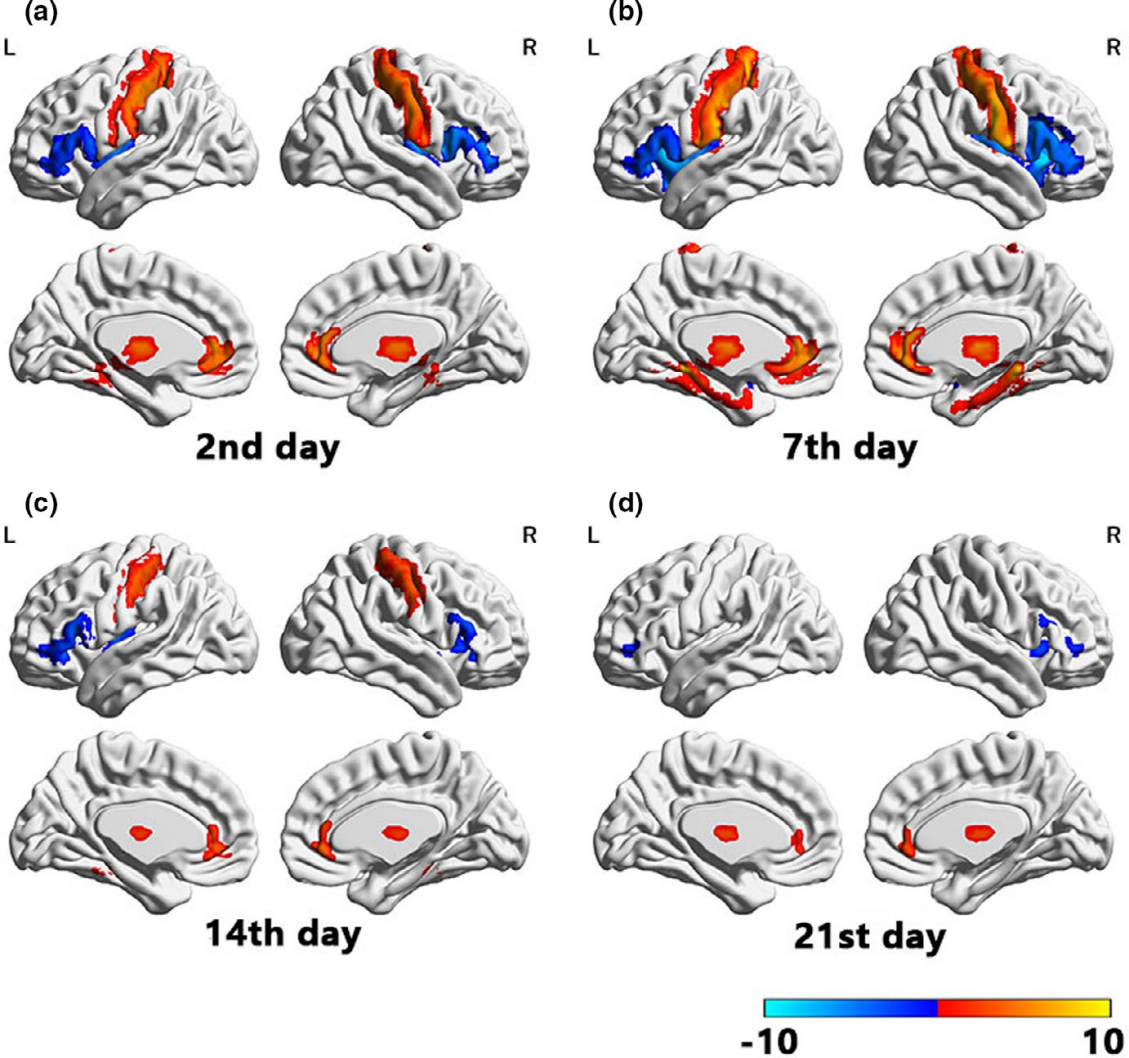
Changes in global functional connectivity density (gFCD) over three weeks of treatment with multi‐infusion ketamine augmentation

At a double threshold of ≥30 clump size and FWE‐corrected *p*‐value of <.001, decreased gFCD values were observed in the bilateral insula, right caudate nucleus, and bilateral inferior frontal gyrus, along with increased gFCD values in the bilateral postcentral gyrus, subgenual anterior cingulate cortex, bilateral thalamus, bilateral parahippocampal gyrus, and anterior lobule of the cerebellum. Ketamine‐induced alterations in brain function increased gradually beginning after the first day of ketamine plus antidepressant treatment, peaking at day 7, and decreasing progressively (Figure 2a–d). These effects had mostly disappeared by the end of the third week.

We observed no significant correlations between changes in HAMD scores and changes in gFCD for any brain region.

## 4 DISCUSSION

To the best of our knowledge, the present study is the first to describe the effects of ketamine augmentation treatment and the associated changes in brain function in patients with TRBPD. Although we found that HAMD scores decreased by nearly 50% after starting ketamine augmentation, these effects were maintained for only one week. Interestingly, the severity of depressive symptoms relapsed from the second week after initiating ketamine treatment, as compared to the baseline, suggesting that ketamine augmentation is not an ideal strategy for combating TRBPD.

More notably, in this pilot study, we observed two remarkable phenomena. First, in patients with TRBPD, the clinical effect of ketamine augmentation was rapidly attenuated, thereby weakening the synergistic effect of ketamine. We postulate that this phenomenon may be related to the rapid desensitization effects of ketamine‐related neurotransmitters (Furuya et al., 1999; Glasgow, Povysheva, Azofeifa, & Johnson, 2017). However, this hypothesis is also limited as one week is a relatively short window of desensitization. If patients with TRBPD exhibit such rapid desensitization in the clinic, it may be necessary to monitor the tendency toward addiction, as previous studies have noted that even low doses of ketamine are associated with an increased risk of addiction (Bonnet, 2015). A previous study reported the clinical experience of using intranasal ketamine in the longitudinal treatment of juvenile patients with BPD who had the “fear of harm” phenotype (Papolos et al., 2018).

Secondly, we found that functional alterations remained apparent even after clinical effects had disappeared, although the *t*‐value was attenuated. Changes in brain function did not completely align with the clinical effects of ketamine augmentation. These findings indicate that the clinical effects are not synchronized with the functional brain alterations found in patients. We postulate that this asynchrony phenomenon may be explained as follows. Although ketamine augmentation induced sustained alterations in brain function, electrical activity induced by ketamine augmentation may have been reduced prior to the attenuation of changes in functional connectivity. The clinical effect is usually associated with the electrical activity of neurons, and interactions among neurons in the brain depend on action potentials (Lyons, Ammari, Hellysaz, & Broberger, 2016; Ma et al., 2017), while fMRI data are based on blood‐oxygen‐level‐dependent (BOLD) signals. Hence, the asynchronous phenomena may be explained by the delay between changes in electrical activity and changes in BOLD signals. However, this hypothesis is limited in that the time delay between changes in electrical activity and BOLD signals exceeded one week. Thus, further studies are required to elucidate the mechanisms underlying this asynchrony.

In the present study, patients with TRBPD exhibited increases in symptom severity relative to the baseline at the end of the study period. Such increases may have been related to the rapid exhaustion of glutamate stores. Previous studies have indicated that ketamine induces rapid glutamate release (Abdallah, Sanacora, Duman, & Krystal, 2015; Weckmann et al., 2019; Wilkinson et al., 2019; Hashimoto et al., 2016; Romeu‐Mejia et al., 2019; Ashok et al., 2017; Xu et al., 2013; Eastwood et al., 2010; Jun et al., 2014), which may explain its rapid antidepressant effects. However, due to the long‐term treatment with mood stabilizers and antidepressants, the glutamate system may become hypoactive in patients with TRBPD (Ballard et al., 2018; Jun et al., 2014). Thus, these patients may not exhibit adequate levels of glutamate for release, even after repeated injections of ketamine, resulting in the relapse of depressive symptoms. Based on our pilot findings, we found that the antidepressant effects of multi‐infusion ketamine infusions in patients with TRBPD rapidly weaken, which may be related to the “neural desensitization” or “glutamate exhaustion.” However, this is only our conjecture and further studies to clarify our hypothesis.

## 5 LIMITATIONS

The present study possesses several limitations of note, including the use of self‐comparisons, which do not provide enough evidence to explain the neural mechanisms underlying the effects of ketamine augmentation on brain function. In addition, the study period was only three weeks, which limited our ability to examine the long‐term dynamics of ketamine plus antidepressant treatment. We were also unable to evaluate the effects of other administration protocols, such as once per day for one week or ketamine plus one week of ECT. Hence, long‐term cohort studies are required to elucidate the trajectory of changes in brain function and the clinical effects of ketamine plus antidepressant treatment. In addition, we were unable to regress out the influence of mood stabilizers and antidepressants, due to the lack of a uniform conversion equation. Although we did fix the dosage and duration of ketamine augmentation, we could not fully avoid the influence of previous pharmacological agents.

While we excluded patients with suicidal ideation, previous reports have shown that ketamine can reduce suicidal ideation in patients with BPD and major depressive disorder (MDD; Abdallah, Sanacora, et al., 2015; Ashok et al., 2017; Ballard et al., 2018; Eastwood et al., 2010; Hashimoto et al., 2016; Ismaylova et al., 2018; Jun et al., 2014; Lyons et al., 2016; Rafael et al., 2015 Soares, Marques, Magalhães, Santos, & Sousa, 2017; Weckmann et al., 2019; Wilkinson et al., 2019; Xu et al., 2013). Future studies should include both BPD and MDD patients to verify this effect. Finally, ketamine can decrease functional activity in the default mode network (DMN) and other brain regions in patients with treatment‐resistant depression (Baldessarini, Vázquez, & Tondo, 2020; van Eijndhoven et al., 2013; Evans et al., 2018; Hirschfeld, 2014; Smith et al., 2018; Subramaniam et al., 2018; Timbie et al., 2015). However, we found that gFCD alterations were not associated with the brain regions commonly detected in previous studies, which focused on the antidepressant effects of ketamine in patients with depression. We postulated that this seemingly contradictory finding might be related to two reasons. First, it may be related to the neuropathological features of BPD, as the neuropathological features of depressive symptoms in patients diagnosed with MDD have been reported to differ from those of depressive symptoms in BPD patients (Baldessarini et al., 2020; Lei et al., 2018). The second reason is related to the functional alteration assessment index. While previous findings have assessed alterations in functional connectivity strength, our pilot findings were based on functional connectivity numbers (Lei et al., 2018), which may explain the inconsistency in altered brain regions between the previous studies and our pilot study.

Next, our “neural desensitization” or “glutamate exhaustion” postulations were conjectured based on a single open‐label pilot study. Moreover, the gFCD alterations in our pilot study require further investigation. According to the findings of Kraus et al., ketamine did not induce significant differences in global brain connectivity before or after ketamine treatment (Kraus et al., 2020). More notably, Gass et al. reported that the influence of repeated treatment on brain topology does not suggest an antidepressant efficacy. Hence, the stability of gFCD alterations induced by multiple ketamine infusion in TRBPD patients requires further investigation.

In this pilot study, we were unable to determine the uniform equivalent dose of antidepressants and diazepam as there is no standard dose conversion formula for antidepressants. However, we fixed the dosages of pharmacological agents across the duration of ketamine augmentation. By comparing before and after ketamine augmentation treatment, we observed the clinical and brain functional alterations. Although the strength of this method was less than that of a double‐blind, randomized controlled study, we believe our findings provide important insight into the mechanism and clinical effects of ketamine augmentation treatment in patients with TRBPD.

## 6 CONCLUSIONS

In this open‐label trial, multi‐infusion ketamine augmentation could rapidly alleviate depressive symptoms in patients with TRBPD. While the clinical effects were not sustained for more than one week, the corresponding functional alterations sharply decreased from the end of the second week. Most of the brain alterations disappeared by three weeks after initiating treatment. Based on these findings, we inferred that the clinical efficacy and functional brain alterations induced by ketamine augmentation are transient.

## CONFLICTS OF INTEREST

None declared.

## AUTHOR CONTRIBUTIONS

CZ, JZ, and HT planned the study. CC, FJi, GC, LW, and FJia enrolled the patients. CC, JZ, XL, DJ, and HT analyzed the data and drafted the manuscript. CZ, JZ, and HT developed the statistical methods and analyzed the data. All authors contributed to interpreting the results, critically evaluating the data, and writing the manuscript.

## Data Availability

The datasets generated and analyzed during the present study are available from the corresponding author on reasonable request.
